# Multi-function screening of probiotics to improve oral health and evaluating their efficacy in a rat periodontitis model

**DOI:** 10.3389/fcimb.2023.1261189

**Published:** 2023-11-07

**Authors:** Qingqing Nie, Xuchun Wan, Hua Tao, Qianqian Yang, Xueyang Zhao, Haixia Liu, Jun Hu, Yanan Luo, Ting Shu, Ran Geng, Zhijing Gu, Fengkai Fan, Zhi Liu

**Affiliations:** ^1^ Department of Biotechnology, College of Life Science and Technology, Huazhong University of Science and Technology, Wuhan, China; ^2^ Institute of Microbiology, Chinese Academy of Sciences, Beijing, China; ^3^ College of Life Sciences, Henan Normal University, Xinxiang, Henan, China; ^4^ Huayuan Biotechnology Institute, Beijing, China; ^5^ National Engineering Research Center for Nanomedicine, College of Life Science and Technology, Huazhong University of Science and Technology, Wuhan, China; ^6^ Hubei Jiangxia Laboratory, Wuhan, China; ^7^ Hubei Key Laboratory of Purification and Application of Plant Anti-Cancer Ingredients, College of Chemistry and Life Science, Hubei University of Education, Wuhan, China

**Keywords:** oral health, oral malodor, probiotics, periodontitis, tooth caries

## Abstract

The oral cavity is the second most microbially rich region of the human body, and many studies have shown that there is a strong association between microorganisms and oral health. Some pathogenic bacteria produce biofilms and harmful metabolites in the mouth that may cause oral problems such as oral malodor, periodontitis, and dental caries. Altering the oral microbiota by using probiotics may alleviate oral health problems. Thus, using multi-function screening, we aimed to identify probiotics that can significantly improve oral health. The main parameters were the inhibition of pathogenic bacteria growth, inhibition of biofilm formation, reduction in the production of indole, H_2_S, and NH_3_ metabolites that cause halitosis, increase in the production of H_2_O_2_ to combat harmful bacteria, and co-aggregation with pathogens to prevent their adhesion and colonization in the oral cavity. Tolerance to cholic acid and choline was also assessed. *Bifidobacterium animalis* ZK-77, *Lactobacillus salivarius* ZK-88, and *Streptococcus salivarius* ZK-102 had antibacterial activity and inhibited biofilm production to prevent caries. They also improved the oral malodor parameter, H_2_S, NH_3_, and indole production. The selected probiotics (especially *L. salivarius* ZK-88) alleviated the inflammation in the oral cavity of rats with periodontitis. The analysis of the gingival crevicular fluid microbiome after probiotic intervention showed that *B. animalis* ZK-77 likely helped to restore the oral microbiota and maintain the oral microecology. Next, we determined the best prebiotics for each candidate probiotic in order to obtain a formulation with improved effects. We then verified that a probiotics/prebiotic combination (*B. animalis* ZK-77, *L. salivarius* ZK-88, and fructooligosaccharides) significantly improved halitosis and teeth color in cats. Using whole-genome sequencing and acute toxicity mouse experiments involving the two probiotics, we found that neither probiotic had virulence genes and they had no significant effects on the growth or development of mice, indicating their safety. Taking the results together, *B. animalis* ZK-77 and *L. salivarius* ZK-88 can improve oral health, as verified by *in vivo* and *in vitro* experiments. This study provides a reference for clinical research and also provides new evidence for the oral health benefits of probiotics.

## 1 Introduction

There are over 10 times more microorganisms living in different parts of the human body than the number of human cells (Berg, 1996). Many studies have shown a strong association between these microorganisms and human health. In particular, intestinal microbial disorders may lead to many diseases such as obesity, degenerative neuropathy, and diabetes (De Vos et al., 2022). The oral cavity, which is a conduit connecting the outside world to the body’s digestive and respiratory tracts, is the second most microbially rich region of the body, with the intestinal tract being the first (Paster et al., 2006). The complex structure of the oral cavity provides diverse habitats for microorganisms with different growth condition requirements (Mark Welch et al., 2020). Some anaerobic microorganisms can colonize the gingival sulcus, while areas with easier access to air, such as the tooth surfaces, tongue, and oral mucosa, are favorable for aerobic microorganisms.

As in the intestines, probiotics may exert beneficial effects on the oral cavity and affect its microorganisms (Homayouni Rad et al., 2023). The oral microbiota and probiotics have become important research subjects (Gungor et al., 2015; Homayouni Rad et al., 2023). Some clinical studies have suggested that there is a strong correlation between *Streptococcus mutans* and caries (Prince et al., 2022). Therefore, researchers have tried to prevent caries by limiting *S. mutans* colonization of the oral cavity (Zhang et al., 2020b; Gu et al., 2022). Due to the ability of the oral microbiota and the host immune system to inhibit the proliferation of exogenous microorganisms (despite the oral cavity often being contaminated with food and oral cleaning products), oral microbial homeostasis can generally be maintained (Li et al., 2019). However, when oral homeostasis is disrupted due to dietary habits, smoking, antibiotics, and pathological factors, the oral dysbiosis may cause a variety of oral problems, such as caries, oral malodor, fungal infection, and periodontitis (Oba et al., 2020; Sivamaruthi et al., 2020). Thus, maintaining the oral microecological balance is of great importance to oral health in humans. Several studies have shown that probiotics may help to maintain oral microbiota homeostasis and improve oral health (Zhang et al., 2020b; Van Holm et al., 2023). Probiotics can colonize the oral cavity and replace certain pathogenic bacteria (Bonifait et al., 2009; Rossoni et al., 2018). They can produce antimicrobial substances (such as fatty acids, H_2_O_2_, and bacteriocins) to resist colonization by pathogenic bacteria (Yoon et al., 2018; Van Holm et al., 2023). Additionally, some probiotics may help to resist the development of low pH in the mouth (Meurman and Stamatova, 2007; Güngör et al., 2013). Considering the oral health-related functions of probiotics, screening for probiotics that can significantly improve the oral microecology may have great promise for disease prevention (Homayouni Rad et al., 2023).

**Figure 1:**
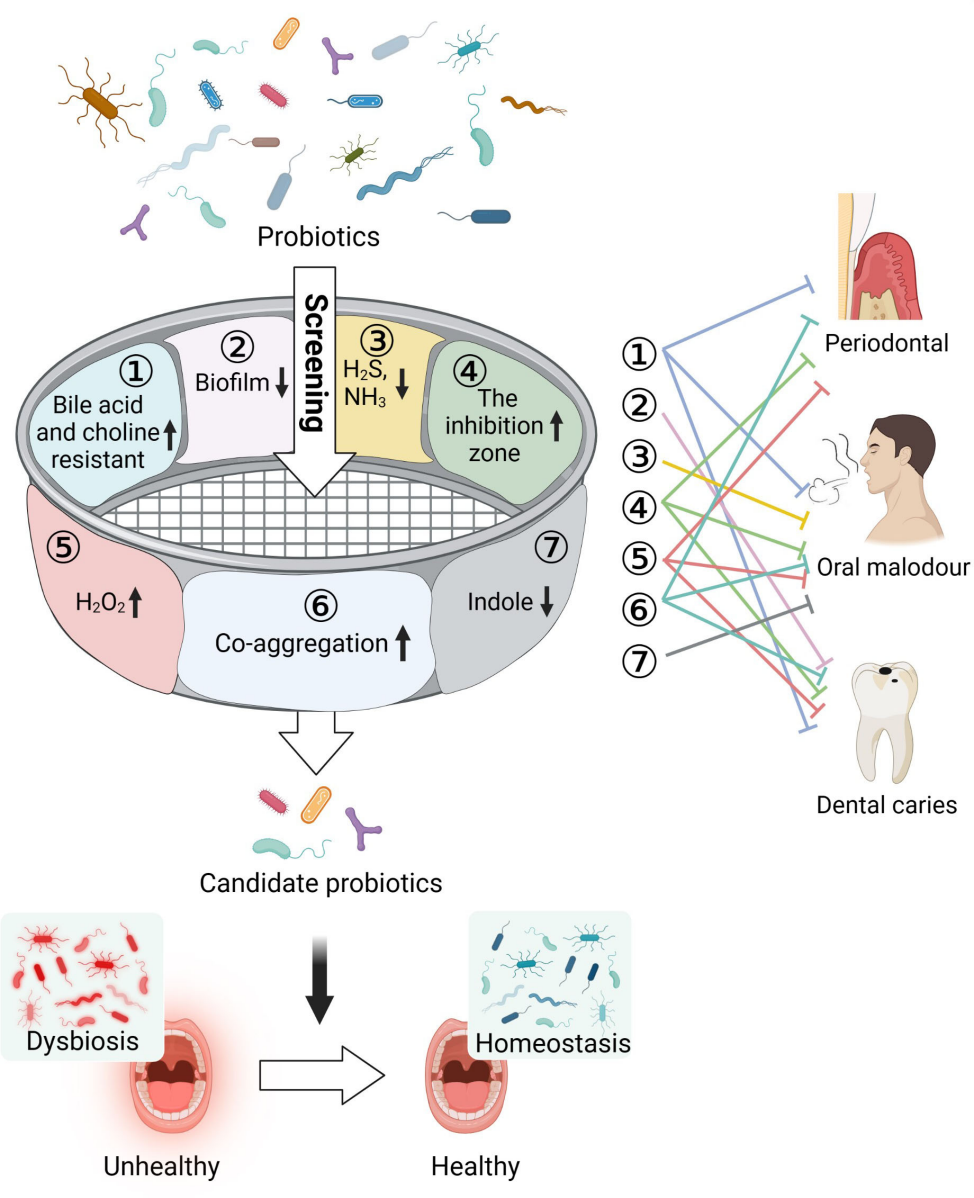
Multi-function screening of probiotics for improving oral health. Oral pathogens can cause a variety of oral problems, such as periodontitis, dental caries, and oral malodor. In total, 48 probiotics were screened based on inhibition of pathogenic bacteria growth, inhibition of biofilm formation, reduction in the production of indole, H_2_S, and NH_3_ metabolites that cause halitosis, increase in the production of H_2_O_2_ to combat harmful bacteria, and co-aggregation with pathogens to prevent their adhesion and colonization in the oral cavity. Tolerance of cholic acid and choline was also assessed. Three probiotics were found to be capable of restoring the oral microecological environment and improving oral health. Created with Biorender.

In this study, using multi-function screening, we identified several probiotics, including *L. salivarius* ZK-88 and *B. animalis* ZK-77, that reduced various parameters that cause oral health problems including H_2_S, NH_3_, and biofilm formation (**Figure 1**). We also conducted *in vivo* experiments in rats using these probiotics to investigate their effects on periodontitis, cytokines, and the microbial community of gingival crevicular fluid. *L. salivarius* ZK-88 and *B. animalis* ZK-77 were combined with a prebiotic and administered to domestic cats to verify the effects. We found that the halitosis and teeth color of the domestic cats were significantly improved. Finally, a preliminary evaluation of the safety of the two probiotics was conducted by whole-genome sequencing and acute toxicity experiments in mice.

## 2 Materials and methods

### 2.1 Strains and culture conditions

Oral probiotics, along with their culture conditions listed in **Supplementary Table S1**, were isolated and preserved in our laboratory, and the potential pathogenic bacteria including *Prevotella copri* CNGBCC1802008 (*P. copri*), *Porphyromonas gingivalis* PG (*P. gingivalis*), *Fusobacterium nucleatum* ATCC25586 (*F. nucleatum*), *Streptococcus mutans* XS30M7 (*S. mutans*), *Escherichia coli* NCTC12900 (*E. coli*), *Staphylococcus aureus* BNCC186158 (*S. aureus*), *Candida albicans* BNCC337321 (*C. albicans*), and *Pseudomonas aeruginosa* PAO1 (*P. aeruginosa*) used in this study were purchased and preserved in our laboratory.

*Lactobacillus* and *Bifidobacterium* were grown in MRS medium (HuanKai Microbial) at 37°C without agitation in an anaerobic environment (80%N_2_, 10%H_2_, 10%CO_2_) for 24hr. *Streptococcus* were grown aerobically in tryptic soy broth (TSB, HuanKai Microbial) at 37°C for 24hr. *F. nucleatum* and *P. gingivalis* were grown anaerobically in brain heart infusion broth (BHI broth, OXOID) supplemented with 0.1% L-Cysteine hydrochloride monohydrate (Sangon Biotech) and 10 µm/mL chlorohemin (Solarbio, IC1380) at 37°C for 72 h. *S. mutans* were grown aerobically in BHI broth supplemented with 10 µm/mL chlorohemin at 37°C for 24 h. *E. coli*, *S. aureus*, *C. albicans*, and *P. aeruginosa* were grown aerobically in LB broth at 37°C for 16 h.

All the strains were inoculated on growth medium agar under aerobic/anaerobic conditions at 37°C. A solitary colony was picked into the growth broth and incubated at 37°C for a suitable time respectively. After subculturing twice, the inoculum was adjusted to an OD_600_ of 1.0 (~10^9^ CFU/mL) using their growth medium for bacterial suspension.

The 5% bacterial suspension was inoculated in MRS broth, modified MRS broth with cholic acid added to adjust the pH to 2.0, and MRS broth containing 0.3% choline salt, respectively. The viable cell counts were measured after 2 hours of treatment.

Isomalt, fructooligosaccharide, xylitol, mint powder, mango juice powder, resistant dextrin, inulin, isomitose oligosaccharides, onion extracts, xylooligosaccharide, and galactooligosaccharide (Guangzhou Zhier Chuangyan Biotechnology Co., Guangzhou Hengyuan Biotechnology Co.) were dissolved in distilled water at a concentration of 0.05g/mL each and filtered through a 0.22um filter membrane to create the prebiotic solution. The bacteria suspension was diluted 20 times with growth medium and 10% prebiotic solution was added. The same bacteria diluent without prebiotics was used as the control. After 24 hours of culture, the bacteria in each sample were detected for comparison.

The Transwell co-culture system was used to evaluate the interaction of two bacterial strains. A 0.22-μm chamber was used to establish a co-culture system of strains A and B so that they would not migrate through the polycarbonate membrane. The A and B were inoculated in the upper and lower chambers respectively. The upper layer was inoculated with a 200 μL suspension of A (inoculated 5%), the lower layer was inoculated with a 700 μL suspension of B (inoculated 5%), and the lower layer was inoculated with a 700 μL blank medium of B as the control (Segura Munoz et al., 2022).

### 2.2 *In vitro* co-aggregation assay

We assessed the co-aggregation of 48 probiotics with four oral pathogens (*P. copri*, *P. gingivalis*, *F. nucleatum*, *S. mutans*). Equal volumes of probiotic suspension and oral pathogen suspension were mixed and incubated at 37°C for 30 min with agitation at 110 rpm. Each probiotic suspension and oral pathogen suspension was also incubated alone at 37°C for 30 min with agitation at 110 rpm. Thereafter, the suspensions were statically cultured at room temperature for 3 min. Next, the absorbance at 600 nm of 200 μL of each culture supernatant was assessed using a microplate reader (Spark 10M, Tecan). The co-aggregation rate (%) was calculated as follows (Keller et al., 2011; Jang et al., 2016):


Co−aggregation rate (%)=[(Aprobiotics+Apathogenic bacteria)÷2−Amix]]÷[(Aprobiotics+Apathogenic bacteria)÷2]×100

A_probiotics_ is the absorbance at 600 nm of the probiotic suspension;

A_pathogenic bacteria_ is the absorbance at 600 nm of the oral pathogen suspension;

A_mix_ is the absorbance at 600 nm of a mix of the oral pathogen and probiotic suspensions.

### 2.3 *In vitro* hydrogen peroxide production assay

Each probiotic suspension was centrifuged at 5000 rpm and 4°C for 10 min. The supernatant was neutralized (pH 7.0) with NaOH and filtered through a 0.45-µm membrane. Next, 100 µL filtrate was assayed using an H_2_O_2_ colorimetric assay kit (Abcam, Cambridge, MA, USA, ab102500). The absorbance at 570 nm was assessed using a microplate reader (Spark 10M, Tecan) in a 96-well enzyme-linked immunosorbent assay microplate (Rowan-Nash et al., 2019).

### 2.4 *In vitro* antibacterial activity assay

The inhibition of pathogenic bacteria by the probiotics was assessed using the double-layer agar method. To create double-layer agar plates, blank solid medium (1.5% agar) was poured into plates and solidified, and then semi-solid medium (0.75% agar) containing 1% pathogen suspension (*P. copri*, *P. gingivalis*, *F. nucleatum*, *S. mutans*, *E. coli*, *S. aureus*, *C. albicans*, *P. aeruginosa*) was poured into the plates to create an upper layer. A 1-mL pipette tip was used to punch holes in the double-layer agar plates. To create probiotic agar blocks to place into the holes, 1 mL of the probiotic suspension was added to 10 mL solid MRS medium (1.5% agar) cooled to 50°C, shaken, poured into a 60-mm petri dish, and anaerobically incubated at 37°C overnight, and then a 1-mL pipette tip was used to obtain probiotic agar blocks to place in the double-layer agar plates. The plates were then incubated overnight at 37°C to assess the inhibitory effects on the pathogens (Kõll-Klais et al., 2005; Keller et al., 2011; Zhang et al., 2020a; Stašková et al., 2021).

### 2.5 *In vitro* antibiofilm formation assay

The probiotic suspensions and pathogen suspensions (*P. copri*, *P. gingivalis*, *F. nucleatum*, *S. mutans*) were diluted 20 times with their corresponding growth medium. Next, 200 µL of probiotic diluent or MRS broth (control) was added to the upper chambers of a 24-well transwell plate, and 1 mL of pathogen diluent was added to the lower chambers. After incubation at 37°C for 48 h, the lower chambers were washed with sterilized distilled water, air-dried for 10 min, stained for 15 min with 1 mL 0.1% crystal violet aqueous solution (BBI, E607309-0100), and washed twice with sterile water. Next, the biofilm formed on the side of each chamber was dissolved in 1 mL dimethyl sulfoxide (DMSO) and the absorbance at 570 nm was assessed using a microplate reader (Spark 10M, Tecan) (Jang et al., 2016; Schwendicke et al., 2017; Zhang et al., 2020a; Stašková et al., 2021).

### 2.6 *In vitro* assays for hydrogen sulfide, ammonia, and indole production inhibition

Each probiotic suspension was filtered through a 0.22-µm membrane and mixed with an oral pathogen suspension at a proportion of 20%. An equal volume of MRS broth was used in place of the probiotic suspension as a control. After anaerobic incubation at 37°C for 48 h, the gas in each anaerobic tube was removed with a syringe. The H_2_S and NH_3_ levels were measured using an H_2_S and NH_3_ detector (BH-90, Bosean) (Jang et al., 2016). Additionally, the culture was centrifuged at 8000 rpm and 4°C for 5 min and 2 mL of the supernatant was evenly mixed with 1.2 mL Ehrlich Reagent (Solarbio, G1290), heated in a water bath at 40°C for 10 min, and then rapidly cooled with running water. The absorbance at 564 nm of 200 μL of the mixture was then assessed using a microplate reader (Spark 10M, Tecan).

### 2.7 Rats and experimental procedures

For the rat experiments, 25 specific-pathogen-free 6-week-old healthy male Sprague–Dawley rats, weighing 180 ± 20 g, were individually maintained in ventilated cages. They were kept under controlled environmental conditions: room temperature (21 ± 2°C), 40 ± 5% humidity, and a 12-h light/dark cycle, with a total pathogen-free diet and water ad libitum.

After 3 days of adaptive feeding, 20 rats were randomly selected and anesthetized by intraperitoneal injection of 300 mg/kg 10% chloral hydrate (Graves et al., 2008; Messora et al., 2013). The remaining 5 rats were used as the normal control (N) group. To establish a periodontitis model using the 20 anesthetized rats, the bilateral maxillary second molars were ligated with absorbable medical silk thread (4-0), which was placed into the gingival sulci as far as possible. If the thread fell off, ligation was performed again. After ligation, the whole mouth was coated every other day with *P. gingivalis* (absorbance at 600 nm = 0.8–1) in a 2% sodium carboxymethyl cellulose solution. The rats were fed a high-sugar diet (Keyes diet 2000 (Saffar et al., 1981), Nantong Teluofei Feed Technology Co. LTD. The ingredients include starch, powdered milk, sugar, wheat flour, yeast, protein powder, and salt).

On day 14 of modeling, the rat periodontitis model was confirmed based on the periodontal probing depth (PPD) index. Next, to create probiotic suspensions, *B. animalis* ZK-77, *L. salivarius* ZK-88, and *S. salivarius* ZK-102 were cultured anaerobically at 37°C overnight, and 100 mL of the culture was centrifuged and then resuspended in 100 mL saline. Thereafter, the 20 rats with periodontitis were randomly assigned to four groups (n=5/group): group M (periodontitis model group) was fed normal saline, group M+B was fed *B. animalis* ZK-77, group M+L was fed *L. salivarius* ZK-88, and group M+S was fed *S. salivarius* ZK-102. Group N (normal control group) received no treatment. The rats were fed the probiotic suspension every 2 days while being fed a normal diet.

### 2.8 Euthanasia and collection of plasma

On day 14 of the probiotic intervention, the rats were euthanized with a lethal dose (150 mg/kg) of sodium thiopental. Then, 500 μL of tail venous blood was collected in a 1.5-mL centrifuge tube pretreated with heparin, kept at room temperature for 30 min, and then centrifuged at 5000 rpm for 2 min. The upper plasma layer was stored at -80°C until testing.

### 2.9 Cytokine measurements

IL-6, IL-10, MCP-1, IFN-γ, tumor necrosis factor (TNF), and IL-12p70 concentrations in the plasma samples were measured using a BD™ Cytometric Bead Array (CBA) Mouse Inflammation Kit (cat. no. 552364, BD Biosciences) according to the manufacturer’s instructions. Briefly, a 50 μL sample was mixed with 50 μL of mixed capture beads and 50 μL of mouse detection reagent. After incubation at room temperature for 2 h in the dark, the samples were washed, suspended in 300 μL of wash buffer, and assessed using a CytoFLEX LX Flow Cytometer (Beckman Coulter, USA). At least 10,000 events were assessed per sample. The data were analyzed using CBA analysis software (FCAP Array, BD Biosciences). The concentration of each cytokine was calculated using a corresponding standard curve.

### 2.10 Periodontitis assessment and micro-computed tomography analysis

On days 1, 3, 5, 7, and 14 of the intervention, the PPD index, bleeding on probing (BOP), and weight of each rat were assessed.

On day 14 of the intervention, alveolar bone specimens were fixed in 4% paraformaldehyde for 24–48 h and sent for section-based scanning by computed tomography imaging (360° rotation with a spatial resolution of 39 µm), and 3D images were reconstructed.

### 2.11 16S rRNA sequencing

After 2 weeks of probiotics intervention, 1 mL of saline was injected into the mouths of the rats and then the fluid was sucked out and immediately frozen at -80°C. The fluid was later subjected to 16S rRNA sequencing. Genomic DNA from the gingival crevicular fluid from the different groups was extracted by CTAB or SDS and PCR amplified with primers for the 16S V4 region (515F and 806R). Library construction was done using the TruSeq^®^ DNA PCR-Free Sample Preparation Kit library building kit and sequencing using Novaseq PE250. After obtaining the raw data, the reads were spliced using FLASH to obtain the raw Tags data (Raw Tags); the assembled Raw Tags need to be strictly filtered to obtain high-quality Tags data (Clean Tags). Referring to the Tags quality control process in Qiime, we performed the following operations: a) Tags intercept: the first low-quality base site of Raw Tags from continuous low-quality value (default quality threshold is <=19) bases to a set length (default length value is 3); b) Tags length filtering: Tags data set obtained after Tags interception, further filter out Tags with continuous high-quality base length less than 75% of the Tags length. After the above treatment, the chimera sequences need to be removed from the Tags. The Tags sequence is compared with the Gold database by UCHIME Algorithm, and the chimera sequences are finally removed to obtain the final valid data (Effective Tags). All Effective Tags were clustered using the Uparse software. By default, the sequences were clustered as OTUs (Operational Taxonomic Units) with 97% consistency. Meanwhile, the representative sequences of OTUs were selected. According to the algorithm principle, the sequence with the highest frequency in OTUs was selected as the representative sequence of OTUs. Species annotation of OTUs and species annotation analysis were done by the Mothur method with the SSUrRNA database (set threshold of 0.8~1), taxonomic information was obtained, and the community composition of each sample was counted at each taxonomic level: kingdom, phylum, class, order, family, genus, and species. Phylogenetic relationships with all OTUs were obtained using the PyNAST software for rapid phylogenetic alignment with the “Core Set” data information in the GreenGene database. Finally, the data of each sample were homogenized, using the standard of the least amount of data in the sample.

### 2.12 Cats and experimental procedures

For the cat experiments, 30 cats drank a solution of *B. animalis* ZK-77 and *L. salivarius* ZK-88 (5×10^9^ CFU/mL) combined with 10% fructooligosaccharides solution at regular intervals every, using a syringe or feeder to slowly inject into the interdental space behind the canine. Out of these 30 cats, 26 had varying degrees of halitosis (degree 1-5), all 30 cats exhibited different levels of yellow teeth, and among them, 13 cats showed redness and swelling (inflammation). Each cat’s mouth was observed on days 0, 7, 14, and 21 and scored as described below.

### 2.13 Scoring halitosis and teeth color in cats

On day 21 of the intervention, each cat’s oral odor was assessed from a distance of 10 cm at intervals of >30 min (Iwamoto et al., 2010; Matsumoto et al., 2018). The organoleptic scoring (Yaegaki and Coil, 2000) was as follows: 0, absence of odor, where none can be detected; 1, questionable odor, where odor is detectable although the examiner could not recognize it as malodor; 2, slight malodor, where odor is deemed to exceed the threshold of malodor recognition; 3, moderate malodor, where malodor is definitely detected; 4, strong malodor, where strong malodor is detected but can be tolerated by the examiner; 5, severe malodor, where overwhelming malodor is detected and cannot be tolerated by the examiner (examiner instinctively averts their nose). Tooth color cards (Vita) were used to assess the tooth color before and on day 21 after the intervention.

### 2.14 Whole-genome sequencing and acute toxicity testing of *B. animalis* ZK-77 and *L salivarius* ZK-88 in mice

Whole-genome sequencing of *L. salivarius* ZK-88 and *B. animalis* ZK-77 was conducted. After obtaining the whole genome second-generation sequencing data of the two strains, we first quality-controlled the raw sequencing reads using the FastQC software and filtered the data according to the report content of the web page. Later, the raw sequencing data were filtered using fastp software to remove the low-quality reads and adapter to obtain clean data. The clean data obtained by fastp filtering was used for sequence splicing directly. Using megahit to perform sequence splicing to obtain the contigs. After obtaining contigs, we used ABRicate to predict the upper virulence factors and antibiotic resistance genes of the genome according to the VFDB and CARD databases and used Prokka to rapidly annotate the prokaryotic genome. To annotate the function of the protein-coding genes, we used the blastKOALA server to annotate CDS by aligning with the Kyoto Encyclopedia of Genes and Genomes (KEGG) database. In addition, we also used the eggNOG-mapper to annotate CDS by aligning with the Clusters of Orthologous Groups (COGs) database.

For the mouse experiments, each probiotic suspension was centrifuged at 3520×*g* for 10 min, resuspended, and washed three times with phosphate-buffered saline, and adjusted with sterile saline to approximately 1×10^10^ CFU/mL. Specific-pathogen-free adult C57BL/6 mice (18 males and 18 females) were acclimatized for 3 days under controlled environmental conditions: room temperature (21 ± 2°C), 40 ± 5% humidity, and a 12-h light/dark cycle, with a total pathogen-free diet and water ad libitum. The mice (6 males and 6 females per group) were randomly divided into a saline gavaged group (control group), an *L. salivarius* ZK-88 gavage group, and a *B. animalis* ZK-77 gavage group (Fan et al., 2023). Each mouse was gavaged with 0.2 mL solution. On days 0, 7, 14, and 21 of the intervention, body weight, behavioral characteristics, poisoning, and death were assessed (Liu et al., 2020).

### 2.15 Statistical analysis

For multi-function screening of each of the 48 probiotics, each of the seven parameters (inhibition of pathogenic bacteria growth, inhibition of biofilm formation, reduction in the production of indole, H_2_S, and NH_3_ metabolites that cause halitosis, increase in the production of H_2_O_2_ to combat harmful bacteria, co-aggregation with pathogens to prevent their adhesion and colonization in the oral cavity, and tolerance to cholic acid and choline) was meticulously evaluated and scored (1–5) using a standardized system, ensuring that each parameter was given the same level of importance. The scores were then summed to determine the total score for each probiotic.

The data are expressed as mean ± SEM. Comparisons between two groups were performed using unpaired two-tailed Student’s t-tests. Comparisons between more than two groups were performed using two-way analysis of variance (ANOVA). Differences were considered statistically significant at *P <* 0.05.

## 3 Results

### 3.1 Probiotics can prevent oral pathogen adhesion and biofilm formation

To investigate whether probiotics can prevent the adhesion and biofilm formation of oral pathogens, we selected several parameters to assess, including H_2_O_2_ production, co-aggregation rate, pathogenic bacterial growth, and pathogenic biofilm formation (**Supplementary Tables S2-1, S2-2, S2-3**, **S2-4**). Among the 48 studied probiotics, the most H_2_O_2_ was produced by *B. animalis* ZK-77 (0.008 μmol/10^4^ cells) followed by *B. longum* ZK-10 (0.0003 μmol/10^4^ cells). The highest co-aggregation rate was exhibited by *L. salivarius* ZK-88 with *P. copri* (7.27%) and *P. gingivalis* (7.48%), by *S. salivarius* ZK-102 with *F. nucleatum* (9.29%), and by *B. animalis* ZK-77 with *S. mutans* (1.61%). Regarding the inhibition of pathogenic bacterial growth, *L. salivarius* ZK-88 and *B. animalis* ZK-77 exhibited the most extensive and potent antibacterial activity. *L. salivarius* ZK-88 and *S. salivarius* ZK-102 showed the strongest inhibition against *P. gingivalis*, with inhibition zone diameters of 40 and 34 mm, respectively. Regarding biofilm formation inhibition, *B. longum* ZK-10 exhibited the highest effect (66%) on *P. copri*, *S. salivarius* ZK-102 (62%) on *P. gingivalis*, *L. salivarius* ZK-88 (69%) on *S. mutans*, and *B. animalis* ZK-77 and *S. salivarius* ZK-102 (31%) on *F. nucleatum*. Overall, based on the above parameters, *L. salivarius* ZK-88, *B. animalis* ZK-77, *S. salivarius* ZK-102, and *L. plantarum* ZK-105 had the best ability to prevent adhesion and biofilm formation of oral pathogens.

### 3.2 Probiotics can inhibit harmful metabolites produced by pathogens

To screen for probiotics that inhibit the harmful metabolites produced by oral pathogenic bacteria, which may aggravate halitosis, we assessed the production of H_2_S, NH_3_, and indole by oral pathogens after probiotic intervention (**Supplementary Tables S2-5, S2-6, S2-7**). First, *B. animalis* ZK-77 and *B. longum* ZK-10 exhibited the highest inhibition of NH_3_ production by *F. nucleatum* (66%) and *P. copri* (97%), respectively. Second, *S. salivarius* ZK-102 and *L. salivarius* ZK-88 exhibited the highest inhibition of H_2_S production by *F. nucleatum* (100%) and *P. gingivalis* (92%), respectively. Lastly, *L. plantarum* ZK-15, *B. animalis* ZK-77, and *S. salivarius* ZK-102 exhibited the highest inhibition of indole production. As good tolerance of probiotics to bile acid and choline may facilitate their beneficial effects, we examined their tolerance. *B. animalis* ZK-140 had the best tolerance, followed by *L. salivarius* ZK-88, *L. plantarum* ZK-105, and *B. longum* ZK-10.

We evaluated the overall performance of the probiotics in preventing the adhesion and biofilm formation of pathogens and inhibiting harmful metabolite production by summing the scores (1–5) of the seven parameters (**Table 1**). The three probiotics with the highest total scores were *B. animalis* ZK-77, *L. salivarius* ZK-88, and *S. salivarius* ZK-102.

**Table 1:** Multi-function screening of potential oral probiotics *in vitro*.

NO.	Strains	Scoring							Scoring sum
		Hydrogen peroxide estimation	Co-aggregation	Antibacterial activity	Inhibition of biofilm formation	Inhibition of H_2_S, NH_3_ production	Inhibition of indole production	Cholic acid and choline tolerance levels	
1	*Bifidobacterium longum* ZK-10	1	1	8	9	8	1	4	32
2	*Bifidobacterium animalis* ZK-13	1	5	12	6	12	3	1	40
3	*Lactobacillus plantarum* ZK-15	1	4	13	7	14	9	1	49
4	*Lactobacillus acidophilus* ZK-16	1	3	13	1	15	2	1	36
5	*Lactobacillus paracasei* ZK-22	1	5	3	4	15	3	0	31
6	*Bifidobacterium animalis* ZK-26	1	3	5	7	7	2	0	25
7	*Lactobacillus plantarum* ZK-27	1	3	6	6	8	3	1	28
8	*Lactobacillus salivarius* ZK-33	1	0	15	6	9	8	2	41
9	*Lactobacillus paracasei* ZK-35	1	4	8	9	8	7	0	37
10	*Streptococcus salivarius* ZK-37	1	0	10	7	14	6	0	38
11	*Bifidobacterium longum* ZK-42	1	3	1	5	13	6	0	29
12	*Bifidobacterium longum* ZK-46	1	1	4	5	10	7	1	29
13	*Bifidobacterium longum* ZK-52	1	1	7	8	7	5	0	29
14	*Bifidobacterium longum* ZK-53	1	4	1	2	7	6	0	21
15	*Lactobacillus salivarius* ZK-64	1	2	7	6	5	7	2	30
16	*Bifidobacterium longum* ZK-65	1	4	8	4	10	8	3	38
17	*Lactobacillus plantarum* ZK-69	1	2	8	4	9	3	1	28
18	*Streptococcus salivarius* ZK-71	1	3	7	6	12	3	0	32
19	*Lactobacillus paracasei* ZK-75	1	3	4	5	11	5	0	29
20	*Bifidobacterium animalis* ZK-77	5	3	21	8	16	9	2	64
21	*Bifidobacterium longum* ZK-79	1	3	15	6	6	2	1	34
22	*Lactobacillus salivarius* ZK-88	1	8	17	9	14	6	4	59
23	*Lactobacillus acidophilus* ZK-89	1	4	11	5	9	4	0	34
24	*Lactobacillus fermentans* ZK-95	1	4	4	6	8	4	0	27
25	*Bifidobacterium adolescentis* ZK-96	1	1	5	3	8	4	0	22
26	*Lactobacillus salivarius* ZK-101	1	3	6	8	10	5	0	33
27	*Streptococcus salivarius* ZK-102	1	5	15	9	15	9	1	55
28	*Bifidobacterium lactis* ZK-103	1	5	2	7	12	3	0	30
29	*Bifidobacterium longum* ZK-104	1	1	2	7	9	7	2	29
30	*Lactobacillus plantarum* ZK-105	1	3	16	6	11	5	4	46
31	*Lactobacillus rhamnosus* ZK-109	1	3	8	7	10	2	0	31
32	*Bifidobacterium longum* ZK-112	1	3	7	6	10	4	0	31
33	*Bifidobacterium longum* ZK-118	1	0	7	5	8	7	1	29
34	*Lactobacillus rhamnosus* ZK-122	1	1	7	7	7	8	0	31
35	*Lactobacillus paracasei* ZK-124	1	4	7	8	11	2	0	33
36	*Lactobacillus salivarius* ZK-125	1	1	7	7	10	5	0	31
37	*Lactobacillus reuteri* ZK-126	1	1	8	3	8	3	0	24
38	*Streptococcus lactis* ZK-128	1	2	4	5	7	1	0	20
39	*Bifidobacterium longum* ZK-129	1	4	1	8	8	6	2	30
40	*Bifidobacterium longum* ZK-131	0	3	1	3	7	4	0	18
41	*Lactobacillus casei* ZK-132	1	4	7	5	14	2	0	33
42	*Bifidobacterium animalis* ZK-140	1	1	13	7	10	6	6	44
43	*Bifidobacterium longum* ZK-141	1	4	7	2	8	7	0	29
44	*Lactobacillus plantarum* ZK-142	1	1	10	5	13	3	1	34
45	*Lactococcus lactis* ZK-143	1	1	8	5	10	4	0	29
46	*Bifidobacterium longum* ZK-144	1	6	4	6	9	6	0	32
47	*Lactobacillus plantarum* ZK-145	1	2	6	6	11	7	1	34
48	*Bifidobacterium longum* ZK-149	1	4	3	5	12	5	0	30

### 3.3 Probiotics can improve the PPD index and alveolar bone resorption

Periodontitis is an inflammatory disease of bacterial etiology, resulting in the loss of periodontal tissue attachment and alveolar bone. The host response to the bacterial challenge leads to clinical signs such as deep pockets, bleeding on probing, gingival recession, and tooth mobility, which can ultimately cause tooth loss (Genco R, 2010).

**Figure 2:**
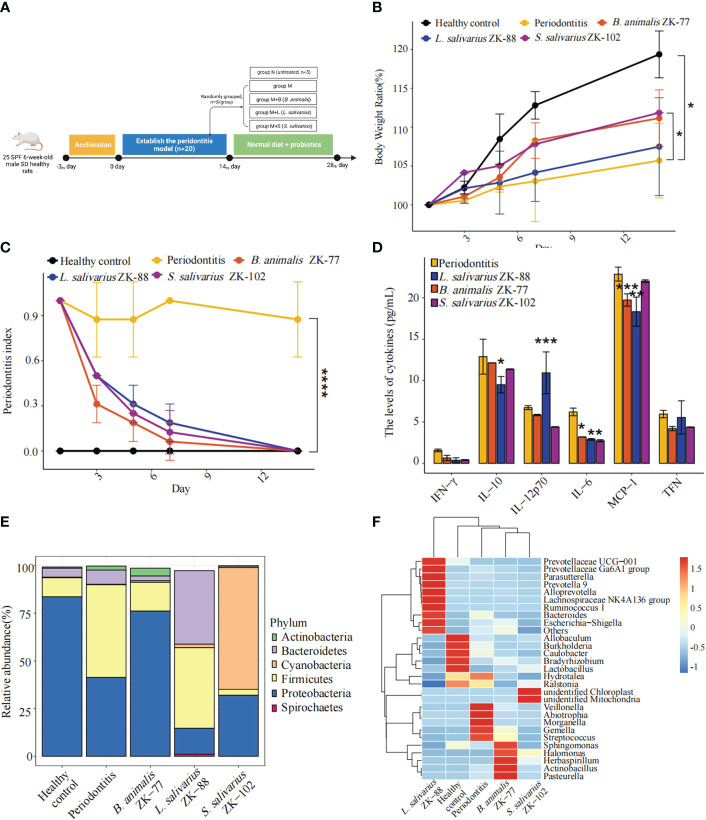
Oral probiotics can improve periodontitis in rats. **(A)** Design of probiotic intervention experiment in rats with periodontitis. **(B)** Changes in body weight. **(C)** Changes in periodontal probing depth (PPD) index. **(D)** Changes in inflammation-related cytokines in plasma. **(E, F)** Changes in the microbial community in gingival crevicular fluid, including a bar plot of phylum-level relative abundances and a heatmap of genus-level relative abundances. All data represent the mean ± SEM. * *P* < 0.05, ** *P* < 0.01, *** *P* < 0.001, **** *P* < 0.0001 vs Periodontitis (two-way ANOVA for **B, C** and Student’s t -tests for **D, E**).

We used a rat periodontitis model to assess whether *B. animalis* ZK-77, *L. salivarius* ZK-88, and *S. salivarius* ZK-102 can improve periodontitis (**Figure 2A**). After the 14-day probiotic intervention, body weight was significantly higher in group M+S than in group M (*P*<0.05, **Figure 2B**). Additionally, the PPD index was significantly lower in groups M+L, M+S, and M+B than in group M (*P*<0.0001, **Figure 2C**), indicating significant improvement in periodontitis.

In group M, compared to group N, alveolar bone resorption (based on 3D optical scanning of the alveolar bone) significantly deteriorated. However, in groups M+B, M+L, and M+S, it was slightly improved by the probiotic intervention (**Supplementary Figure S1**).

### 3.4 Probiotics can regulate inflammation-related cytokines

The periodontal bacterial species *P. gingivalis* alters cytokine expression in gingival epithelial cells, stimulating inflammatory responses that may lead to periodontal disease (Zhao et al., 2012). These periodontal bacteria enter the oral cavity via the blood, oral–pharyngeal, and oral–digestive pathways, leading to various inflammatory diseases (Hajishengallis and Chavakis, 2021). In a previous study of a periodontitis model, there was increased gingival expression of inflammatory mediators, including molecules that are implicated in bone resorption such as interleukin (IL)-6, TNF, and IFN-γ (Hajishengallis et al., 2011). In contrast, macrophages produce IL-12 p70 to clear *P. gingivalis* by inducing T cells and natural killer cells to produce IFN-γ, which in turn activates the bactericidal function of macrophages (Trinchieri, 2003; Hajishengallis et al., 2007).

Plasma inflammatory factors IL-6 (*P*<0.05), IL-10 (*P*<0.05), and MCP-1 (*P*<0.01) were lower in group M+L than in group M, while IL-12p70 (*P*<0.001) was higher (**Figure 2D**). Additionally, IL-6 (*P*<0.05) and MCP-1 (*P*<0.001) were lower in group M+B than in group M, and IL-6 (*P*<0.05) was lower in group M+S than in group M. Thus, *L. salivarius* ZK-88 had the best inhibitory effect on inflammation.

### 3.5 *B. animalis* ZK-77 is beneficial for microbial community recovery

We constructed a bar plot of the top five phyla in each of the five groups based on mean relative abundances in the gingival crevicular fluid microbial community (**Figure 2E**). The microbiota structure was changed in group M compared to group N, with Firmicutes being significantly increased and Proteobacteria being significantly decreased in group M. Interestingly, *B. animalis* ZK-77, *L. salivarius* ZK-88, and *S. salivarius* ZK-102 altered the microbiota. In particular, the microbial composition in group M+B became similar to group N, implying that *B. animalis* ZK-77 normalized the oral microbiota in group M. However, there were no significant similar effects in groups M+S and M+L.

We also constructed a heatmap of the top 10 genera in each of the five groups based on the mean relative abundances (standardized and centralized by Z-scores) in the gingival crevicular fluid microbial community (**Figure 2F**). *Veillonella*, *Abiotrophia*, *Morganella*, *Gemella*, and *Streptococcus* were significantly increased in group M compared to group N, while *Allobaculum*, *Burkholderia*, *Caulobacter*, and *Lactobacillus* were significantly decreased. *Streptococcus mutans* can produce lactic acid, which induces caries (Cai et al., 2021). The heatmap showed that *Streptococcus* was significantly reduced in groups M+B, M+L, and M+S. Overall, *B. animalis* ZK-77 showed good ability for the recovery of the microbial community in rats.

### 3.6 Combined probiotics/prebiotic (*B. animalis* ZK-77, *L. salivarius* ZK-88, and fructooligosaccharides) can improve halitosis and teeth color in cats

**Figure 3:**
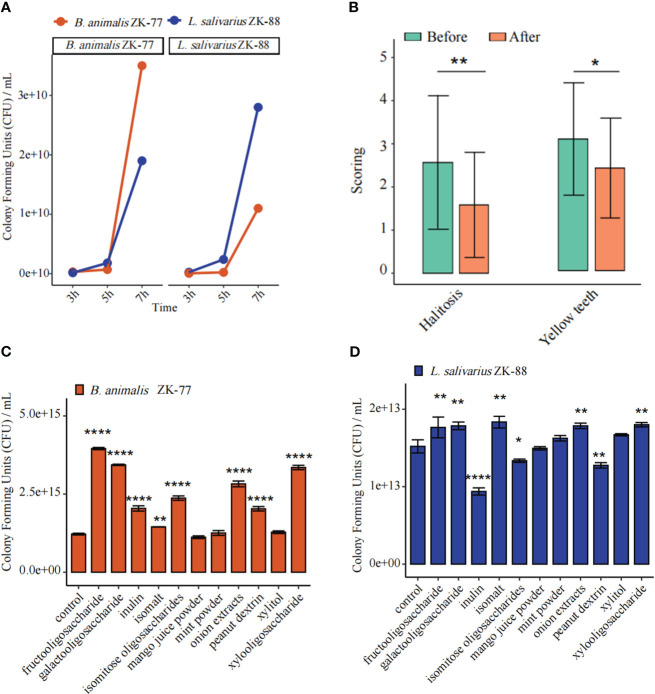
Combined probiotics/prebiotic (*B. animalis* ZK-77, *L. salivarius* ZK-88, and fructooligosaccharides) was validated in cats with oral disease. **(A)** *B. animalis* ZK-77 and *L. salivarius* ZK-88 could coexist according to an *in vitro* coexistence assay. **(B)** Combined probiotics/prebiotic improved halitosis and teeth color in cats. The data represent the mean ± SEM (n=30). * *P* < 0.05, ** *P* < 0.01 vs Before (Student’s t -tests). **(C, D)** Prebiotics promoted the growth of *B. animalis* ZK-77 and *L. salivarius* ZK-88. All data represent the mean ± SEM. * *P* < 0.05, ** *P* < 0.01, **** *P* < 0.0001 vs control (Student’s t-tests).

To investigate whether *B. animalis* ZK-77 and *L. salivarius* ZK-88 can coexist, we conducted an *in vitro* coexistence assay. They were found to coexist well (**Figure 3A**). A prebiotic is “a non-digestible food ingredient that beneficially affects the host by selectively stimulating the growth and/or activity of one or a limited number of bacteria in the colon, and thus improves host health” (Gibson and Roberfroid, 1995).

Next, we co-cultured *B. animalis* ZK-77 and *L. salivarius* ZK-88 with media containing different prebiotics. Fructooligosaccharides promoted the growth of *B. animalis* ZK-77 (*P*<0.0001) (**Figure 3C**), while fructooligosaccharides, isomalt, galactooligosaccharide, onion extracts, and xylooligosaccharide promoted the growth of *L. salivarius* ZK-88 (*P<*0.01) (**Figure 3D**).

The effects of combined probiotics with a prebiotic (*B. animalis* ZK-77, *L. salivarius* ZK-88, and fructooligosaccharides) were validated in 30 cats with oral diseases such as halitosis, and oral redness and swelling (inflammation). After 21 days, the halitosis score decreased from 2.57 to 1.58 (*P*<0.01) and the teeth color score decreased from 3.05 to 2.34 (*P*<0.05). Thus, combined probiotics with a prebiotic can improve halitosis and teeth color in cats (**Figure 3B**; **Supplementary Figure S2**).

Furthermore, we tracked whether there were any adverse reactions and learned from the feedback that the 30 cats did not show any side effects. In total, 13 cats had redness and swelling (inflammation) gums, but nine cats showed no obvious improvement after the probiotic intervention. Three cats showed improvement, with only one showing significant improvement (**Supplement Table S3**). In conclusion, the probiotics composition can significantly improve halitosis and yellow teeth, and improve gingival inflammation in some cats, and it is safe and has no side effects in cats.

### 3.7 Whole-genome sequencing and acute toxicity testing preliminarily reveal the safety of *B. animalis* ZK-77 and *L. salivarius* ZK-88

**Figure 4:**
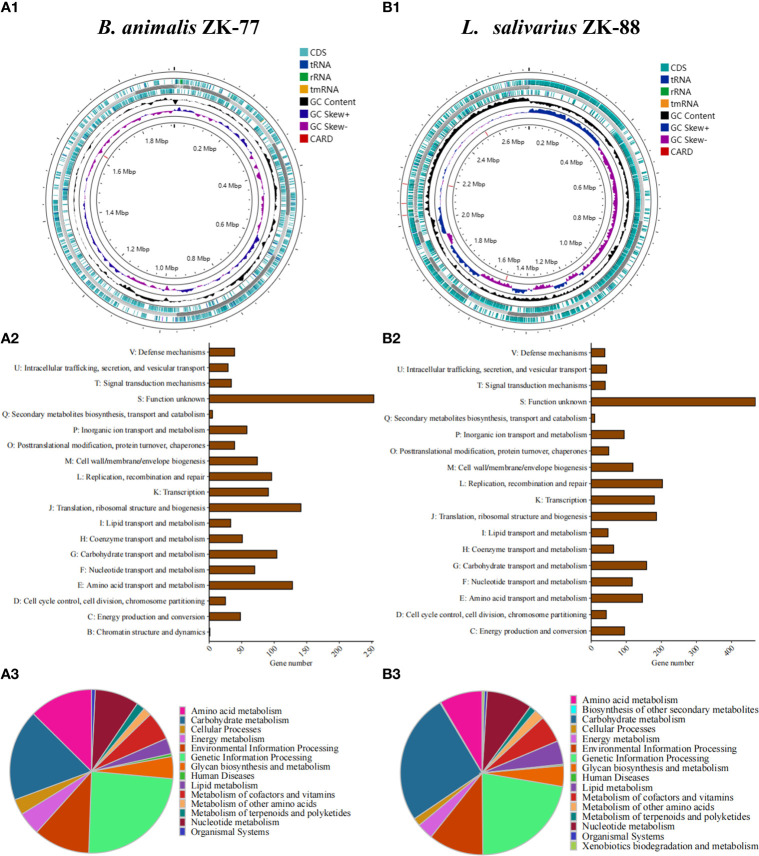
Whole-genome sequencing of *B. animalis* ZK-77 and *L. salivarius* ZK-88. **(A1, B1)** Circular representation of *B. animalis* ZK-77 and *L. salivarius* ZK-88 genomes. From outer to inner rings: CDS with homology to known antimicrobial resistance genes on the forward strand, genes on the forward strand, contigs, genes on the reverse strand, GC content and GC skew, and CDS with homology to known antimicrobial resistance genes on the reverse strand. **(A2, B2)** COG annotation of *B. animalis* ZK-77 and *L. salivarius* ZK-88 genomes. COG functional categories are shown on the y-axis and the number of genes in these COG categories are shown on the x-axis. **(A3, B3)** KEGG annotation of *B. animalis* ZK-77 and *L. salivarius* ZK-88 genomes.

We sequenced the whole genomes of *B. animalis* ZK-77 and *L. salivarius* ZK-88. We annotated 1,558 CDS, 6 rRNAs, 1 repeat region, 61 tRNAs, and 1 tmRNA in *B. animalis* ZK-77 (**Figure 4A1**), and 2,805 CDS, 22 rRNA, 1 repeat region, 90 tRNAs, and 1 tmRNA in *L. salivarius* ZK-88 (**Figure 4B1**). In addition, we annotated the virulence genes and antibiotic resistance genes of the two probiotics using ABRicate. Based on the VFDB database, neither probiotic had virulence genes, indicating their safety. However, based on the CARD database, we found one tetracycline resistance gene in *B. animalis* ZK-77 (**Figure 4A1**), and one tetracycline resistance gene and two aminoglycoside resistance genes in *L. salivarius* ZK-88 (**Figure 4B1**). The impact of these antibiotic resistance genes on safety needs to be further evaluated.

In addition, we conducted KEGG and COG functional annotation analyses of the CDS in the two probiotics. In *B. animalis* ZK-77, the 1,558 CDS were classified into 14 KEGG categories, with the top categories being nucleotide metabolism, environmental information processing, amino acid metabolism, carbohydrate metabolism, and genetic information processing (**Figure 4A3**). The CDS were classified into 19 COG categories, with the top categories being translation, ribosomal structure, and biogenesis (J); amino acid transport and metabolism (E); and carbohydrate transport and metabolism (G) (**Figure 4A2**). In *L. salivarius* ZK-88, the 2,805 CDS were classified into 16 KEGG categories, with the top categories being glycan biosynthesis and metabolism, lipid metabolism, metabolism of cofactors and vitamins, amino acid metabolism, nucleotide metabolism, environmental information processing, genetic information processing, and carbohydrate metabolism (**Figure 4B3**). The CDS were classified into 18 COG categories, with the top categories being replication, recombination, and repair (L); transcription (K); translation, ribosomal structure, and biogenesis (J); carbohydrate transport and metabolism (G); nucleotide transport and metabolism (F); and amino acid transport and metabolism (E) (**Figure 4B2**).

Furthermore, we assessed the safety of *B. animalis* ZK-77 and *L. salivarius* ZK-88 *in vivo* experiments. During the experiment, all groups behaved actively, with normal food intake and water consumption. There was no diarrhea, death, or other disease symptoms. The appearance of the intestinal segments and visceral organs was normal on autopsy, and no obvious pathological changes were seen with the naked eye. As seen in **Supplementary Figure S3**, the mice that were gavaged with *B. animalis* ZK-77 and *L. salivarius* ZK-88 had the same growth pattern as the blank control group, and their body weight increased normally.

Based on the results of whole-genome sequencing and acute toxicity testing, it was preliminarily determined that the two strains are safe for use in animals.

## 4 Discussion

Several studies have revealed that microorganisms may perform beneficial functions in the oral cavity, such as alleviating dysbiosis (Lin et al., 2022), inhibiting biofilm formation (Wu et al., 2015), and treating periodontitis (Teughels et al., 2013). Therefore, we aimed to screen for probiotics that can improve various oral health parameters. Through multi-function screening of 48 candidate probiotics, we identified the two best probiotics (*L. salivarius* ZK-88 and *B. animalis* ZK-77) that can improve various parameters such as oral malodor and inflammation. However, oral cavity problems such as periodontitis may be caused by a complex oral dysbiosis, where various beneficial species (which have various roles and coordinate with each other) are decreased and various pathogenic bacteria are increased, rather than being caused by a single bacterial strain (Hajishengallis and Lamont, 2012; Lamont et al., 2018). This means that monitoring only one or a few pathogenic bacteria as an indicator of disease would be imperfect. Thus, although we comprehensively screened the 48 candidate probiotics and identified two probiotics based on multiple parameters, we still need more elucidation from a microecological perspective as well as more insight into the mechanisms behind the beneficial effects to promote the application of the probiotics. Furthermore, it is notable that the heterogeneity of strains, hosts, and their oral microbiomes may lead to the failure to solve oral problems in different people (Veiga et al., 2020). Therefore, the future development of probiotics should be more customizable.

As prebiotics may enhance the effect of probiotics (Sanders et al., 2019), we formulated two probiotics with a prebiotic to test their benefits in an everyday scenario (that is, for improving the oral health of cats). Despite having identified two beneficial probiotics, this is only a preliminary study. A more optimal choice, which could be more complex or customizable, remains to be identified. Additionally, the complex diet and oral cleaning habits of humans make their oral environment significantly different from that of cats. Therefore, human trials are still needed to further validate the potential of probiotics for improving human oral health. The more drastic external disturbances to which the human oral cavity may be subjected, as well as the lifestyle differences of different human populations, may present additional obstacles to the identification of optimal oral probiotics and the evaluation of their efficacy. Even if future screening yields probiotics for improving oral health in humans, there is a need to develop appropriate processes for their use to ensure that their beneficial functions are not disrupted too greatly by diet or brushing.

Our probiotic screening strategy was based on the effects of probiotics on various oral health parameters, without evaluating the duration of the beneficial effects. It is preferable that the duration is as long as possible while also maintaining a healthy oral microecological balance. Probiotics may interact with other microorganisms in the mouth or become inactive in the presence of food components, thus decreasing the duration of the beneficial effects. Maintaining the viability of the probiotics is one of the critical factors for prolonging this duration. The formulation of probiotics using techniques such as microencapsulation may help to prevent rapid loss of probiotic viability. Overall, the duration of the beneficial effects on the oral microecological balance needs to be further evaluated and this evaluation criterion should be incorporated into subsequent probiotic screening.

## 5 Conclusion

We screened for probiotics that significantly improved oral microecology. The results suggested that *L. salivarius* ZK-88 and *B. animalis* ZK-77 are beneficial for inhibiting pathogenic bacteria from producing harmful metabolites such as H_2_S and NH_3_, and therefore have the potential to improve oral malodor. We designed rat experiments to explore the effects of the probiotics on periodontitis and found that the probiotics significantly improved periodontitis. To enhance the effectiveness of the probiotics, the two best probiotics were combined and a prebiotic was added. Subsequently, we validated the functionality of the combined probiotic formulation in the oral cavity of cats, demonstrating its ability to improve their oral health. Whole-genome sequencing of the two probiotics preliminarily revealed their safety. In conclusion, our study provides new evidence for the application of probiotics in the maintenance of oral health.

## Data availability statement

The datasets presented in this study can be found in online repositories. The names of the repository/repositories and accession number(s) can be found below: https://www.ncbi.nlm.nih.gov/, PRJNA994816 https://www.ncbi.nlm.nih.gov/, PRJNA994833.

## Ethics statement

All animal experiments were approved by the Institutional Animal Care and Use Committee of Huazhong University of Science and Technology ([2020] IACUC Number: 2996). All animals received humane care and experimental protocols were carried out in accordance with the Guide for the Care and Use of Laboratory Animals of Huazhong University of Science and Technology, as approved by the Animal Care Committee of Hubei Province. All animal procedures and experiments reported in this study were performed in accordance with ARRIVE guidelines.

## Author contributions

QN: Conceptualization, Investigation, Writing – original draft, Writing – review & editing, Data curation. XW: Investigation, Visualization, Writing – original draft, Writing – review & editing. HT: Conceptualization, Writing – review & editing. QY: Software, Visualization, Writing – original draft. XZ: Investigation, Methodology, Writing – review & editing. HL: Investigation, Methodology, Writing – review & editing. JH: Validation, Writing – review & editing, Funding acquisition. YL: Investigation, Methodology, Writing – review & editing. TS: Investigation, Methodology, Writing – review & editing. RG: Investigation, Methodology, Writing – review & editing. ZG: Investigation, Methodology, Writing – review & editing. FF: Conceptualization, Project administration, Supervision, Writing – review & editing. ZL: Conceptualization, Funding acquisition, Project administration, Supervision, Writing – review & editing.
