# Supplementary material for: Multi-function screening of probiotics to improve oral health and evaluating their efficacy in a rat periodontitis model
Source: Front Cell Infect Microbiol. 2023 Nov 7;13:1261189. doi: 10.3389/fcimb.2023.1261189 (PMC10660970; doi:10.3389/fcimb.2023.1261189)
Supplement: Supplementary file 1 [file DataSheet_1.docx]

Supplementary materials

# Supplementary Figures


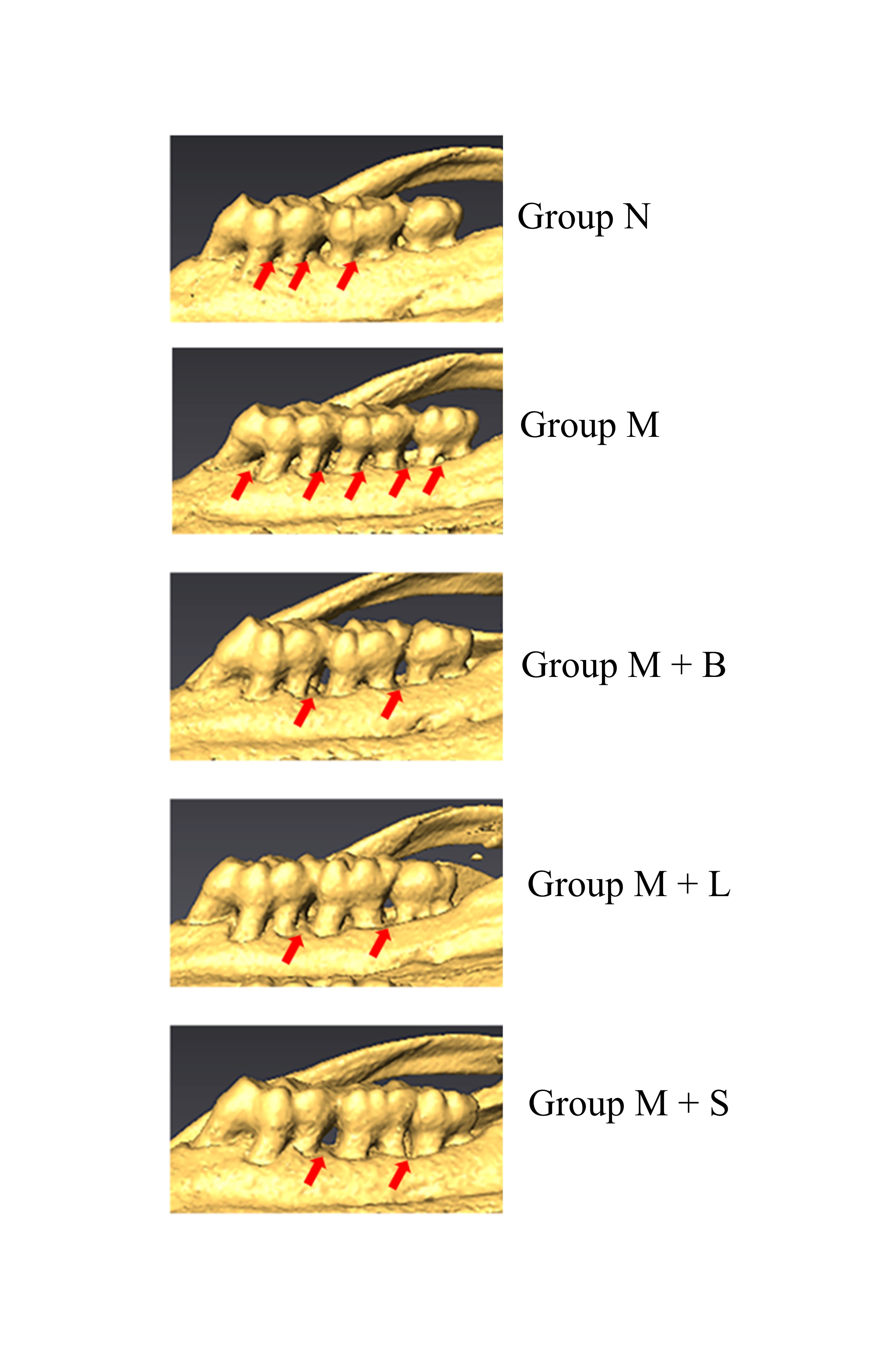


**Supplementary Figure S1 |** 3D scan of alveolar bone resorption in rats. After the probiotic intervention, alveolar bone destruction in groups M+B, M+L and M+S compared to group M showed a tendency to be suppressed.


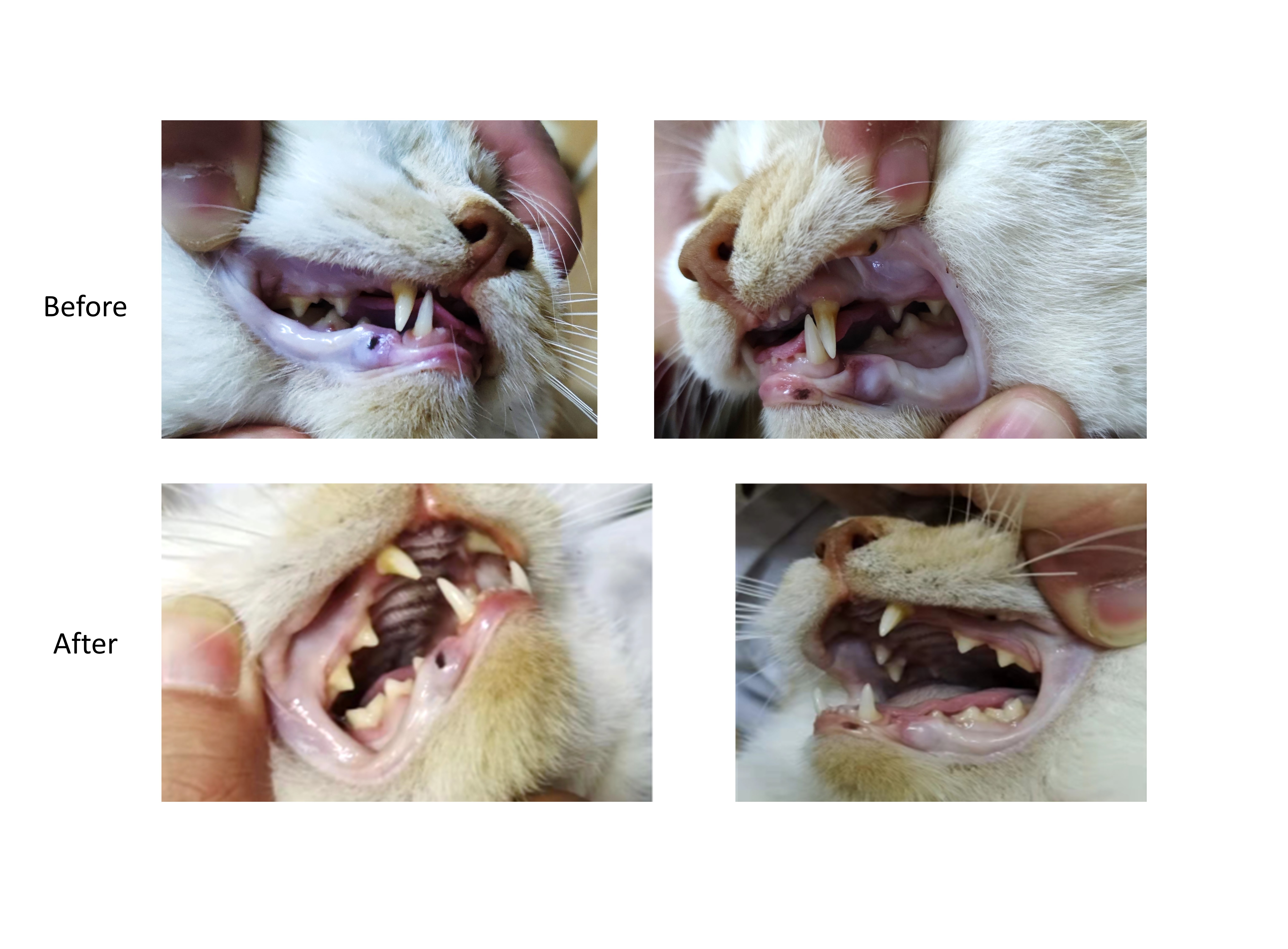


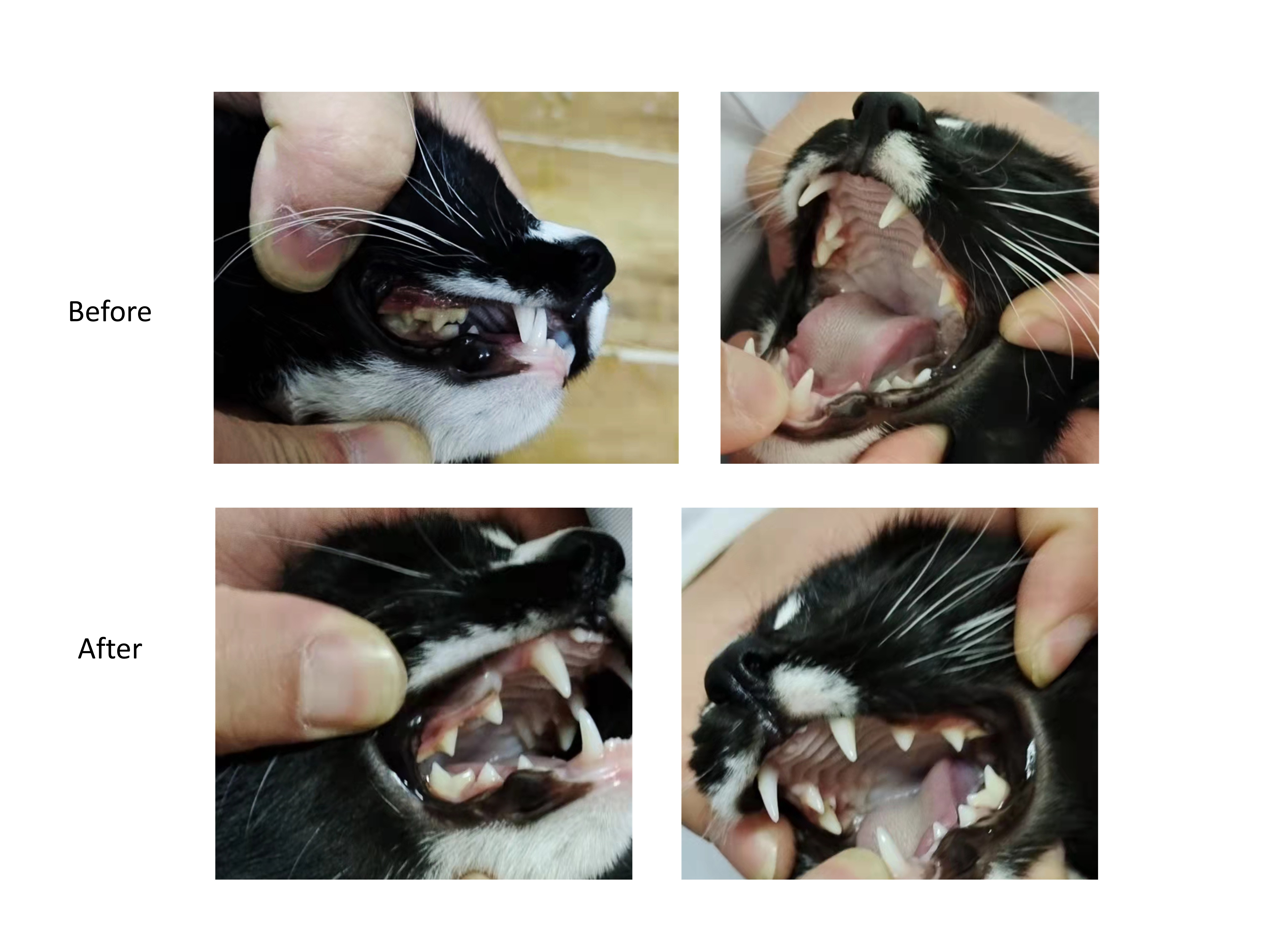


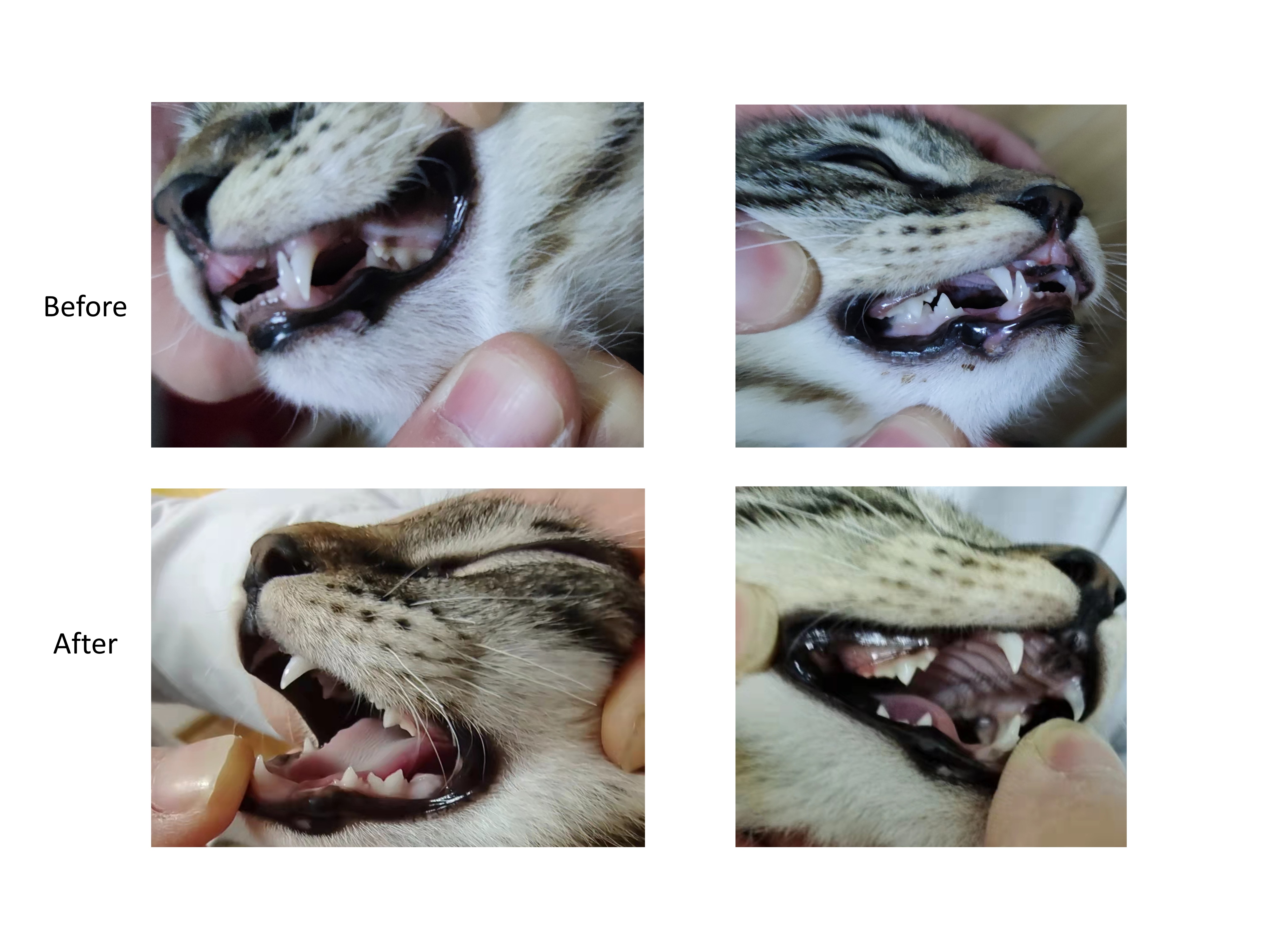


**Supplementary Figure S2 |** Changes in tooth color after probiotic intervention in cats
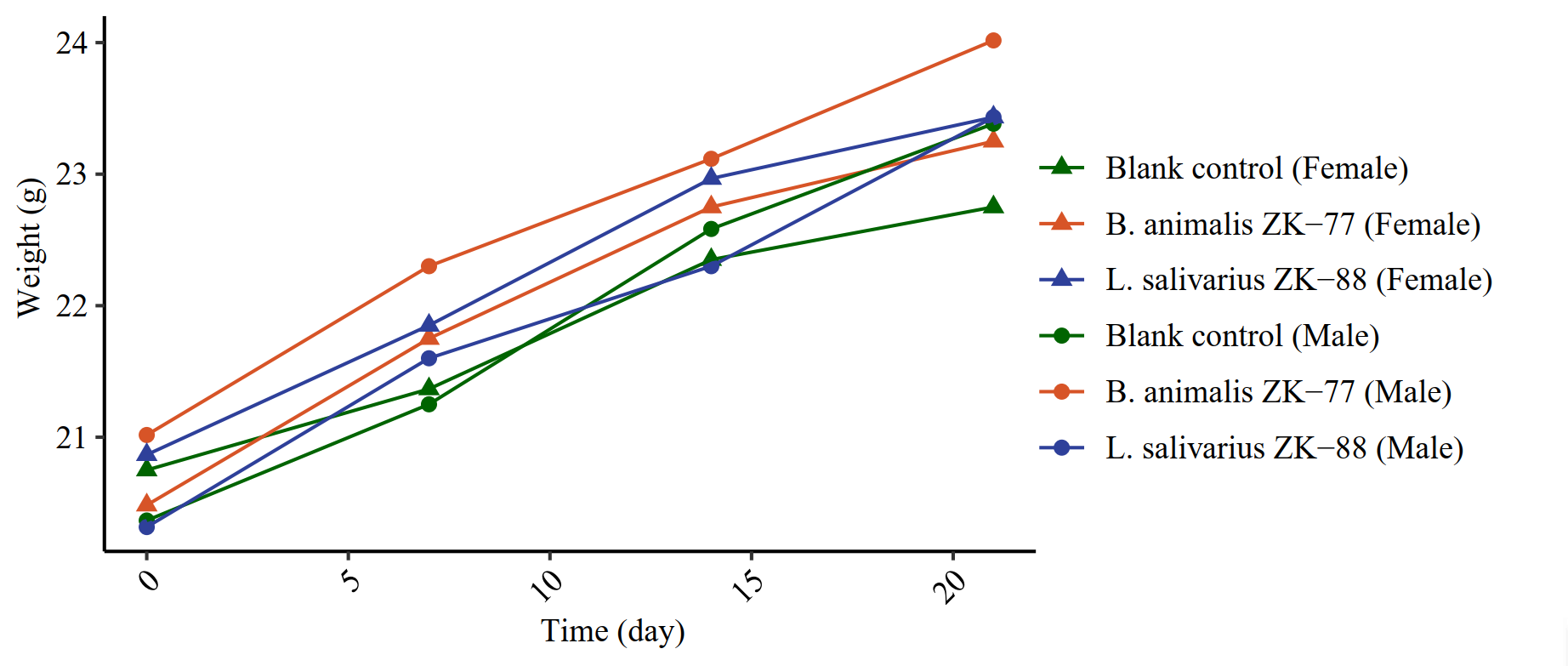


**Supplement Figure S3 |** The weight changes of mice that were gavaged with B. animalis ZK-77, L. salivarius ZK-88 or saline
